# SrfABC Toxin from *Xenorhabdus stockiae* Induces Cytotoxicity and Apoptosis in HeLa Cells

**DOI:** 10.3390/toxins11120685

**Published:** 2019-11-22

**Authors:** Yawei Sun, Guoyong Zhang, Xiaoqing Hou, Shuai Xiao, Xi Yang, Yali Xie, Xiyin Huang, Fan Wang, Xiangtao Mo, Xuezhi Ding, Liqiu Xia, Shengbiao Hu

**Affiliations:** State Key Laboratory of Developmental Biology of Freshwater Fish, Hunan Provincial Key Laboratory for Microbial Molecular Biology, College of Life Science, Hunan Normal University, Changsha 410081, China; syw@smail.hunnu.edu.cn (Y.S.); zhanggy1120@163.com (G.Z.); hxq@smail.hunnu.edu.cn (X.H.); xshnsd@163.com (S.X.); yangxiwang7@163.com (X.Y.); xieyali68@163.com (Y.X.); hxy@smail.hunnu.edu.cn (X.H.); wf@smail.hunnu.edu.cn (F.W.); hsbalp@163.com (X.M.); dingxuezhi@hunnu.edu.cn (X.D.); xialq@hunnu.edu.cn (L.X.)

**Keywords:** SrfABC toxin, cytotoxicity, apoptosis, HeLa cells, co-immunoprecipitation

## Abstract

Our previous study showed that the *srfABC* operon, which was originally identified in *Salmonella enterica* as an SsrB-regulated operon clustered with the flagellar class 2 operon, exhibited significant cytotoxicity against insect midgut CF-203 cells and injectable insecticidal activity against *Helicoverpa armigera* larvae. The *srfABC* operon was widely distributed among bacteria, which raises the question of their biological roles in different species. In this study, we investigated the cytotoxic effect of SrfABC toxin on mammalian cell lines. When simultaneously expressed in the *Escherichia coli* cytoplasm, SrfABC exhibited cytotoxicity against all tested mammalian cancer cell lines (B16, 4T-1, Hep-3B, and HeLa) in a dose-dependent manner. Intracellular expression of SrfA–FLAG, SrfB–FLAG, or SrfC–FLAG also resulted in inhibition of proliferation and apoptosis on HeLa cells. When incubated with HeLa cells separately, SrfA, SrfB, and SrfC proteins alone could enter HeLa cells, then induce apoptosis and cytotoxicity. SrfC protein shifts its localization from cytoplasm to nucleus with the aid of SrfA and/or SrfB protein. Although SrfA, SrfB, and SrfC proteins alone exhibited a cytotoxic effect against HeLa cells, all three components were essential for the full cytotoxicity. Native PAGE and co-immunoprecipitation assay demonstrated that SrfA, SrfB, and SrfC proteins could interact with each other and form a heteromeric complex.

## 1. Introduction

*Xenorhabdus* spp. are symbionts of entomopathogenic nematodes (EPN) of the genus *Steinernema*, where they reside in a special vesicle in infective juveniles (IJ) [1]. Upon finding a suitable host, the nematode physically penetrates the insect and migrates to the hemocoel, while the bacteria are transferred directly into the hemocoel where they are able to evade the insect’s immune response and kill the insect using a variety of toxic mechanisms [2,3]. A wide range of toxins with oral and/or injectable insecticidal activities have been identified and characterized in *Xenorhabdus* spp., such as Xpts (Xenorhabdus protein toxins) [4], XhlA (cell surface-associated hemolysin) [5], XaxAB (Xenorhabdus α-xenorhabdolysin) [6], Txp40 (40 kDa toxin from *Xenorhabdus* and *Photorhabdus*) [7], etc. [8].

In our effort to screen novel toxins from the genomic fosmid library of *Xenorhabdus stockiae* HN_xs01, the FS2 clone, which bears the *srfABC* operon, exhibited excellent cytotoxicity against insect midgut CF-203 cells [9]. The *srfABC* operon was originally identified in *Salmonella enterica* as an SsrB-regulated operon, clustered with the flagellar class 2 operon [10]. In the genome of *X. stockiae* HN_xs01, several genes linked to type VI secretion system (T6SS) post-translational control proteins lie in the neighborhood of the *srfABC* operon. The open reading frames (ORFs) of *srfA*, *srfB*, and *srfC* analogs in *X. stockiae* HN_xs01 were 1395 bp, 3021 bp, and 2682 bp in size, and the theoretical molecular weight (MW) of corresponding encoded proteins was 51.3 kDa, 114.2 kDa, and 101.8 kDa, respectively. Although SrfA, SrfB, and SrfC alone exhibited cytotoxicity against CF-203 cells, all three components of SrfABC toxin were essential for full cytotoxicity. SrfABC toxin significantly induced apoptosis and G2/M phase arrest in CF-203 cells. Furthermore, SrfABC toxin exhibits highly injectable insecticidal activity against *Helicoverpa armigera* larvae [9].

Several tripartite toxins have been extensively studied. Haemolysin BL (Hbl) secreted by *Bacillus cereus* is the first known three-component bacterial toxin and thought to contribute to diarrheal food poisoning and necrotizing infections [11]. Hbl, composed of three components (designated as B, L1, and L2), is a unique membrane-lytic toxin. It was proposed that Hbl B alone might be able to oligomerize on the cell surface and form a pore. L2 and L1, which are definitely required for toxic activity, might either stabilize B, induce conformational changes to B, or even enter the cell [12]. Cytolethal distending toxins (CDTs), another important tripartite toxin, which have been described in several important bacterial pathogens (*Campylobacter jejuni*, *Campylobacter coli*, *Campylobacter fetus*, *Shigella spp.*, *Haemophilus ducreyi*, *Helicobacter hepaticus*, etc.) can cause eukaryotic cells to arrest in the G2/M transition phase of the cell cycle and induce cytoplasm distention and chromatin fragmentation [13,14]. CdtA, CdtB, and CdtC interact with one another to form an active tripartite holotoxin that exhibits full cellular toxicity. CdtB acts as the enzymatically active subunit, while CdtA and CdtC are required for the delivery of CdtB [15,16]. Lately, tripartite toxins with insecticidal activity have also been identified [17]. The Toxin complexes (Tcs) were first identified as high-molecular weight insecticidal complexes present in the *Photorhabdus luminescens* strain W14 [18]. Xpt toxin complex, an analog of Tc, is composed of three different proteins, which can be categorized as class A, B, and C proteins based on sequence similarity and size. XptA2, XptB1, and XptC1, representing class A, B, and C proteins, combined in a respective 4:1:1 stoichiometry. The class A protein forms a 1140 kDa tetramer. The class B and C proteins (XptB1 and XptC1) form a binary complex and strongly bind to the tetrameric XptA2 to form the complete and fully active toxin complex [19].

Although the highest occurrence of the *srfABC* operon was found in members of the family enterobacteriaceae, including *Xenorhabdus*, *Yersinia*, *Pantoea*, *Serratia*, *Dickeya*, *Citrobacter*, *Pectobacterium*, etc., the *srfABC* operon was widely distributed in various bacteria from classes of gammaproteobacteria, alphaproteobacteria, and betaproteobacteria. The wide distribution of the *srfABC* operon in such a range of bacteria raises the question of their biological role in different species. Here, we determined the cytotoxic effect of SrfABC toxin on mammalian cell lines, and its potential mechanism was also investigated.

## 2. Results and Discussion

### 2.1. SrfABC Toxin Exhibits Cytotoxicity Against Several Mammalian Cancer Cell Lines

**Figure 1:**
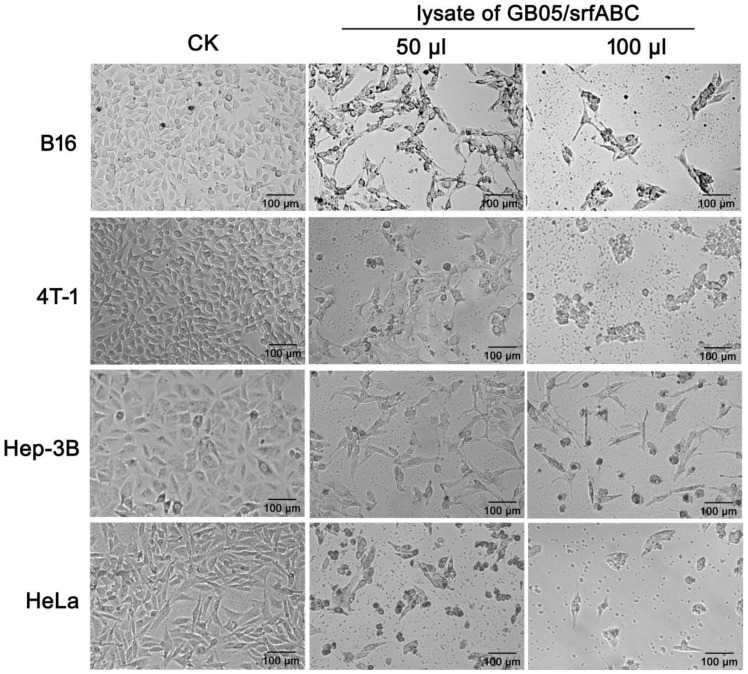
Cytotoxicity of SrfABC toxin against mammalian cancer cells. B16, murine melanoma cells; 4T-1, murine mammary carcinoma cells; Hep-3B, human hepatoma cells; HeLa, human cervical carcinoma cells. In the experimental group, mammalian cancer cells were incubated with 50 μL or 100 μL of L-arabinose-induced lysates of GB05/srfABC for 48 h. Morphological changes of cells were observed under an inverted light microscope.

To determine the cytotoxicity of SrfABC toxin on mammalian cancer cells, murine melanoma cell line B16, murine mammary carcinoma cell line 4T-1, human hepatoma cell line Hep-3B, and human cervical carcinoma cell line HeLa were treated with various amounts of the lysates of GB05/srfABC after being induced with L-arabinose. After 48 h, the proliferation of cancer cells treated with 25 μL of L-arabinose-induced lysates of GB05/srfABC was slightly inhibited (data not shown), while 50 μL lysates greatly inhibited the proliferation of all four cancer cell lines when compared with that of the control (Figure 1). One hundred microliters of L-arabinose-induced lysates of GB05/srfABC treatment could not only enhance the proliferation inhibition effects, but also induce dramatic morphological changes in cancer cells, including cell rounding and cytoplasmic shrinkage. When the dosage of lysates was elevated to 200 μL, cells were hardly observed under a microscope (data not shown). Meanwhile, cells appeared to be normal, both in number and morphology, in the control groups, in which cells were treated with the lysates of GB05/pSC101-BAD-gbaA. SrfABC appeared to be more potent in HeLa cells compared with the other three cell lines. Our previous study showed that all three components of SrfABC toxin could be solubly expressed in GB05/srfABC with the induction of L-arabinose. Taken together, we concluded that when simultaneously expressed in the *Escherichia coli* cytoplasm, SrfABC exhibited cytotoxicity against mammalian cancer cells in a dose-dependent manner.

Several insecticidal toxins from *Photorhabdus* and *Xenorhabdus* have been reported to be toxic against both insect cells and mammalian cells. Tc (PTC3 and PTC5) induced major changes of the actin cytoskeleton in both *Galleria mellonella* hemocytes and HeLa cells [20]. XaxAB-like binary toxin exhibited a cytotoxic effect against both insect cells (CF-203) and mammalian cells (B16, 4T1, and HeLa cells) [21]. Our previous study showed that SrfABC toxin significantly induced G2/M phase arrest and apoptosis in CF-203 cells [9], indicating that SrfABC toxin had an identical type of cell surface receptor and similar type of intracellular target in both insect cells and mammalian cancer cells.

### 2.2. Transient Expression of SrfA, SrfB, or SrfC in HeLa Cells Elicited Cytotoxicity

**Figure 2:**
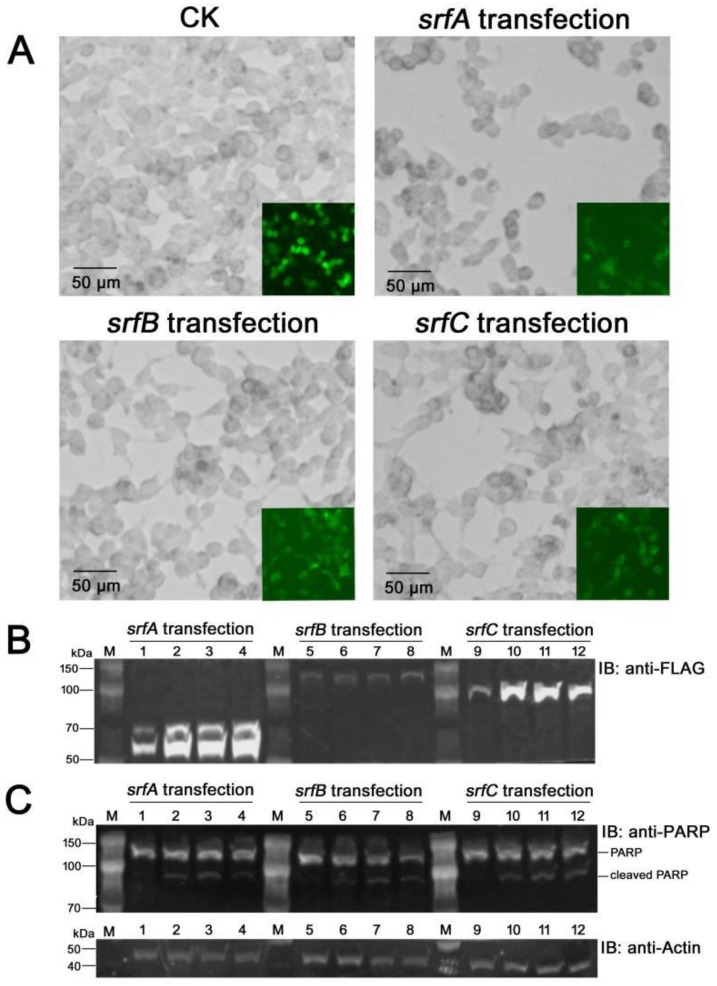
Transient expression of SrfABC toxin components in HeLa cells by transfection. (**A**) Proliferation and morphology of transfected HeLa cells under an inverted light microscope. (**B**) Detection of the expression of SrfA–FLAG, SrfB–FLAG, and SrfC–FLAG in transfected HeLa cells by western blotting using anti-FLAG antibody. (**C**) Detection of the apoptotic cleavage of poly (ADP-ribose) polymerase (PARP) in the transfected HeLa cells by western blot analysis using anti-PARP antibody. CK: HeLa cells transfected with pcDNA5/FRT/TO/FLAG; *srfA* transfection: HeLa cells transfected with pFLAG–SrfA; *srfB* transfection: HeLa cells transfected with pFLAG–SrfB; *srfC* transfection: HeLa cells transfected with pFLAG–SrfC. Lanes 1, 5, and 9: Transfected HeLa cells sampled at 12 h post-transfection; lanes 2, 6, and 10: Transfected HeLa cells sampled at 24 h post-transfection; lanes 3, 7, and 11: Transfected HeLa cells sampled at 36 h post-transfection; lanes 4, 8, and 12: Transfected HeLa cells sampled at 48 h post-transfection. β-actin was used as an internal control to show equal loading of proteins.

As there generally exists an enzymatically active component in tripartite cytotoxins (CdtB component of CDT toxin [15], TcC component of Tc toxin [20]), we then transiently expressed SrfA, SrfB, and SrfC protein in HeLa cells by transfection to assess the cytotoxic effects of each component of SrfABC toxin. The ORFs of *srfA*, *srfB*, and *srfC* genes were amplified by PCR and cloned into the eukaryotic expression vector pcDNA5/FRT/TO/FLAG to create pFLAG–SrfA, pFLAG–SrfB, and pFLAG–SrfC respectively. Three plasmids were independently transfected into HeLa cells, and the expression of FLAG fusion proteins was detected by western blotting using anti-FLAG antibody. All three FLAG fusion proteins, SrfA–FLAG, SrfB–FLAG, and SrfC–FLAG, could be detected in HeLa cells at 12 post-transfection and stably expressed after transfection for 24 h, 36 h, and 48 h (Figure 2B). While both SrfA–FLAG and SrfC–FLAG fusion proteins exhibited high expression levels from 24 h to 48 h of post-transfection, SrfB–FLAG fusion protein was expressed at much lower levels. For SrfA–FLAG fusion protein detection, in addition to the band corresponding to estimated molecular weight of 54 kDa, the antibody also interacted with another higher molecular weight band with an estimated molecular weight of ~70 kDa, which suggested that SrfA–FLAG fusion protein underwent a post-translational modification.

Cellular expression of SrfA–FLAG, SrfB–FLAG, or SrfC–FLAG resulted in inhibition of proliferation of HeLa cells with varying extents when compared to the control cells that were transfected with pcDNA5/FRT/TO/FLAG. In particular, marked morphological changes such as cell shrinkage, rounding, and formation of membrane blebs were observed in the HeLa cells expressing SrfA–FLAG protein (Figure 2A). As these morphological changes were identical to those usually associated with apoptosis, we then detected the apoptotic cleavage of poly (ADP-ribose) polymerase (PARP) in the transfected HeLa cells by western blot analysis using anti-PARP antibody. At 12 h of post-transfection, only a 113 kDa signal could be detected in all three transfected HeLa cell lines. At 24 h of post-transfection, a second signal, typical of apoptosis, appeared at 89 kDa in addition to the 113 kDa signal in all three transfected HeLa cell lines. The 89 kDa signal could still be detected at 48 h of post-transfection (Figure 2C). It is well known that the unique cleavage of PARP is an early hallmark of apoptosis in HeLa cells [22]. The above data indicated that transient expression of SrfA, SrfB, or SrfC alone could induce apoptosis on HeLa cells.

### 2.3. Each Component of SrfABC Toxin-Induced Cytotoxicity and Apoptosis on HeLa Cells

**Figure 3:**
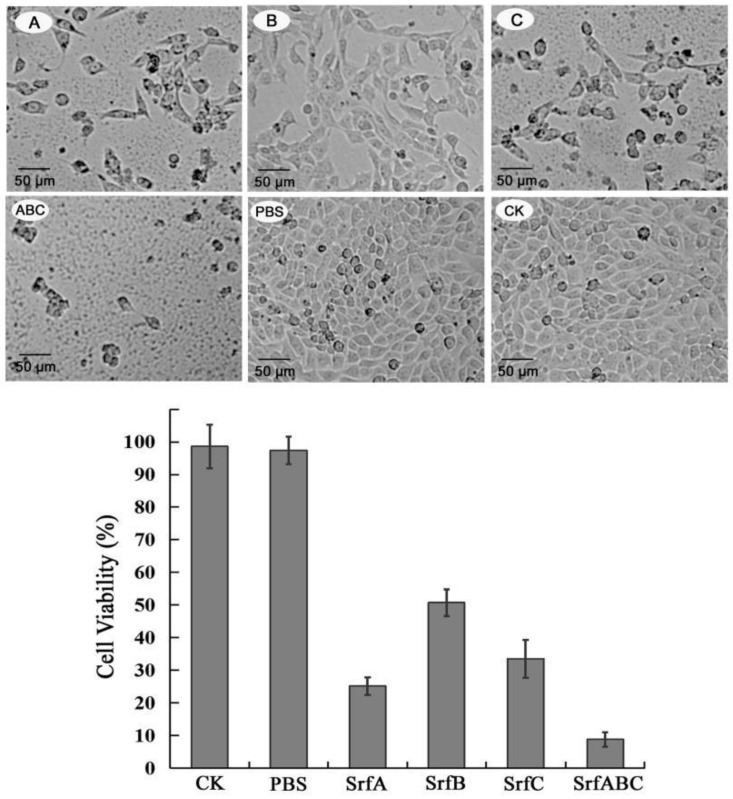
Cytotoxicity of SrfABC toxin against HeLa cells. Upper panel: Morphology of HeLa cells treated with SrfABC toxin. (**A**) Treatment with SrfA protein alone; (**B**) treatment with SrfB protein alone; (**C**) treatment with SrfC protein alone; A + B + C: Treatment with SrfABC mixture; PBS: Treatment with PBS buffer; CK: Untreated HeLa cells. Lower panel: Cell toxicity was measured using an MTT assay after 48 h of exposure to SrfA (150 nM), SrfB (150 nM), or SrfC (150 nM) alone or their mixture (50 nM for each component).

As our previous study showed that each component of SrfABC exhibited cytotoxicity against insect CF-203 cells, we then explored whether SrfA, SrfB, or SrfC alone could also cause toxic effects on HeLa cells. Purified GST-tagged SrfA, SrfB, and SrfC proteins [9] were applied to HeLa cells and incubated for 48 h. MTT assay demonstrated that each component of SrfABC toxin inhibited the viability of HeLa cells. While 150 nM of SrfA, SrfB, and SrfC alone resulted in 72%, 48%, and 66% loss of cell viability, respectively, their combination (50 nM for each component) caused around 90% of HeLa cells to lose viability (Figure 3). Under an inverted microscope, untreated HeLa cells showed a typical fibroblastic appearance and HeLa cells treated by PBS had a morphology similar to that of the control. SrfB protein alone inhibited the proliferation of HeLa cells, while SrfA protein alone or SrfC protein alone could not only greatly inhibit the proliferation of HeLa cells, but also cause morphological changes (cell rounding and cytoplasmic shrinkage). These morphological changes were more obvious when three components were mixed (Figure 3). In agreement with the findings of MTT assay in CF-203 cells, all three components of SrfABC toxin were essential for full cytotoxicity against HeLa cells.

**Figure 4:**
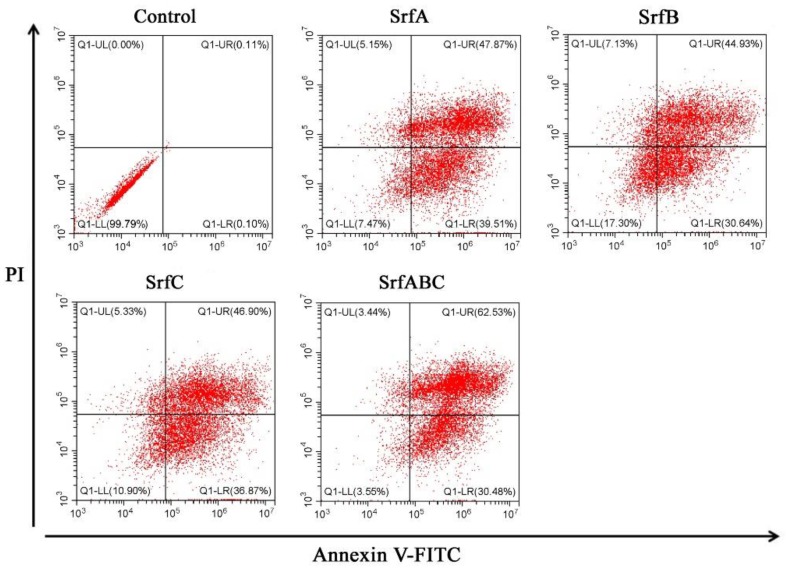
SrfABC toxin-induced apoptosis in HeLa cells. Cells treated with SrfA, SrfB, or SrfC alone or their mixture for 48 h were subjected to flow cytometry analysis. Control: HeLa cells treated with PBS buffer.

We then quantified cellular apoptosis and necrosis by flow cytometric analysis. HeLa cells treated with SrfA, SrfB, or SrfC protein alone or their mixture for 48 h were stained with Annexin V and Propidiumiodide (PI), and analyzed by flow cytometry (Figure 4). The cytogram quadrant of Annexin V-FITC staining positive represents an apoptotic event, and PI staining positive represents a necrotic event. Q1-UL is the region of necrotic event where Annexin V-FITC staining is negative and PI staining is positive. Q1-UR is a late apoptosis event where both Annexin V-FITC and PI staining is positive. Q1-LR is an early apoptosis event with cells stained with Annexin V-FITC alone. Q1-LL represents an event of negative staining for both Annexin V-FITC and PI. The percentage of apoptotic cells at the basal level was 0.21% in untreated HeLa cells. When treated with SrfA, SrfB, or SrfC protein alone, the total percentage of apoptotic HeLa cells (early and late apoptosis) was 87.38%, 75.57%, and 83.77% respectively. The treatment of the mixture of three components resulted in 93.01% apoptotic cells (62.53% early apoptosis, 30.48% late apoptosis). Statistical analysis showed that there existed statistically significant differences between treated groups and the control group (*p* < 0.05).

### 2.4. Confocal Microscopy Revealed Different Intracellular Localizations of SrfABC Toxin to HeLa Cells

**Figure 5:**
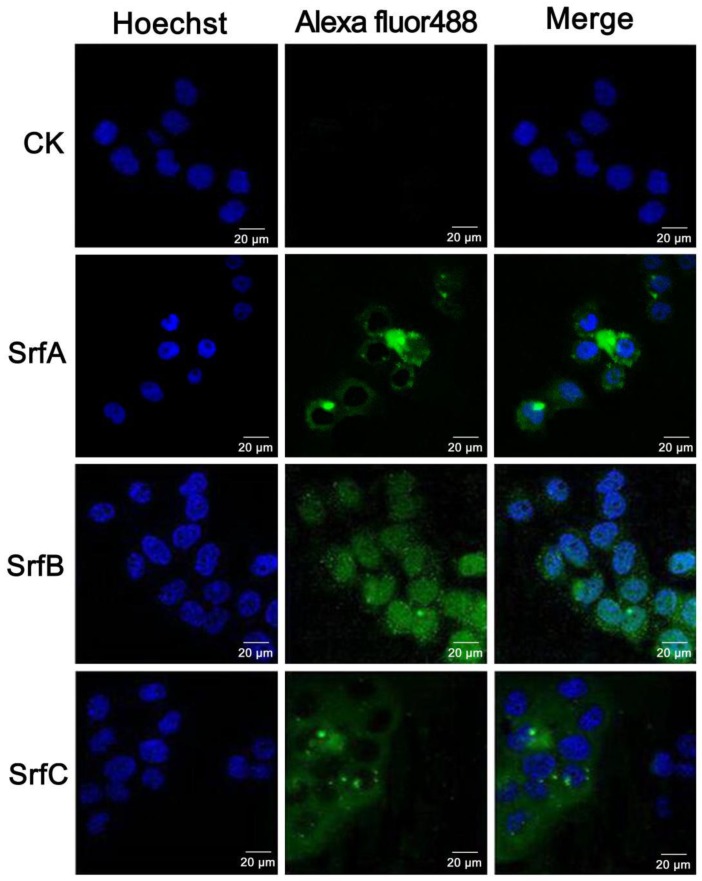
Immunostaining and confocal microscopy analysis of intracellular localizations of SrfABC toxin to HeLa cells when separately applied. HeLa cells treated with SrfA (150 nM; the second panel), SrfB (150 nM; the third panel), or SrfC (150 nM; the fourth panel) protein were fixed with methanol and permeabilized with 0.25% Triton X-100, followed by separately incubating with anti-His–SrfA (the second panel), anti-His–SrfB (the third panel), or anti-His–SrfC (the fourth panel) primary antibodies. Then, cells were stained with Alexa Fluor 488-conjugated secondary antibody and Hoechst 33342. Alexa Fluor 488: The primary antibodies were detected using Alexa Fluor 488-conjugated secondary antibody; Hoechst: The nuclei of HeLa cells was stained with Hoechst 33342. CK: HeLa cells treated with PBS buffer and incubated with anti-His–SrfA, anti-His–SrfB, and anti-His–SrfC primary antibodies.

As SrfA, SrfB, and SrfC protein alone exhibited cytotoxic effects against HeLa cells, we investigated whether SrfA, SrfB, or SrfC protein could enter HeLa cells independently. HeLa cells treated with SrfA (150 nM), SrfB (150 nM), or SrfC (150 nM) protein alone for 48 h were fixed and immunostained with anti-SrfA, anti-SrfB, or anti-SrfC primary antibody, respectively. Alexa Fluor 488-conjugated secondary antibody was used to detect the primary antibody, while Hoechst 33342 was used to stain nuclei. Stained samples were inspected with confocal microscopy. As shown in Figure 5, separately incubated with HeLa cells for 48 h, each component was detected intracellularly; namely, SrfA and SrfC proteins were localized to the cytoplasm, while SrfB protein was localized to the nucleus (Figure 5). Thus, we concluded that each component of SrfABC toxin could enter HeLa cells independently.

**Figure 6:**
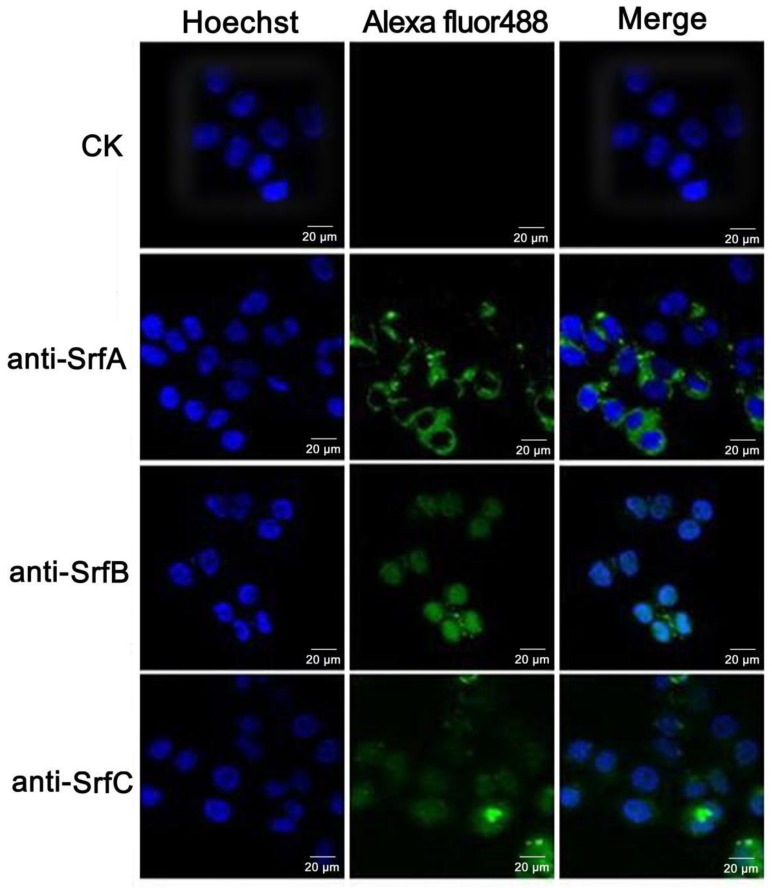
Immunostaining and confocal microscopy analysis of intracellular localizations of SrfABC toxin to HeLa cells when their mixture (50 nM for each component) was applied. HeLa cells treated with SrfABC mixture were fixed with methanol and permeabilized with 0.25% Triton X-100, followed by separately incubating with anti-His–SrfA (the second panel), anti-His–SrfB (the third panel), or anti-His–SrfC (the fourth panel) primary antibodies. Then, cells were stained with Alexa Fluor 488-conjugated secondary antibody and Hoechst 33342. Alexa Fluor 488: The primary antibodies were detected using Alexa Fluor 488-conjugated secondary antibody; Hoechst: The nuclei of HeLa cells was stained with Hoechst 33342. CK: HeLa cells treated with PBS buffer and incubated with anti-His–SrfA, anti-His–SrfB, and anti-His–SrfC primary antibodies.

When three components (50 nM for each component) were mixed together, their intracellular localization could also be detected in HeLa cells; namely, SrfA protein was localized to the cytoplasm, while SrfB and SrfC proteins were localized to the nucleus, i.e., SrfC protein shifts its localization from cytoplasm to nucleus in HeLa cells in the presence of SrfA and/or SrfB protein (Figure 6). The above results indicated that SrfA and/or SrfB protein must play a role in the translocation of SrfC into the nucleus of HeLa cells. It is therefore possible that these three proteins or a subset of them may interact with one another to form a complex prior to delivery of SrfC into the nucleus.

### 2.5. Three Components Could Interact with Each Other and Form a Heteromeric Complex

**Figure 7:**
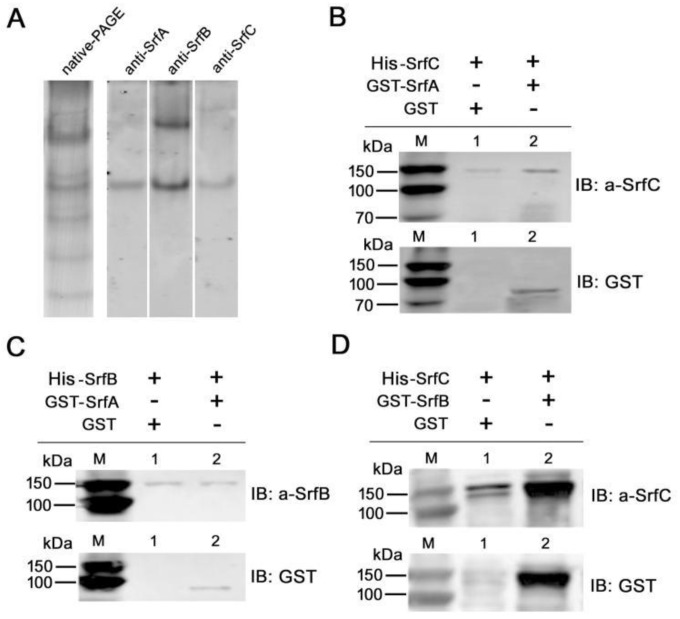
Detection of interaction between each component of SrfABC toxin. (**A**) The lysate of L-arabinose-induced GB05/srfABC was resolved by 6% native PAGE gel and analyzed by western blot using anti-His–SrfA, anti-His–SrfB, or anti-His–SrfC antibodies, respectively. (**B**) Co-immunoprecipitation assays for interaction between SrfC and SrfA. (**C**) Co-immunoprecipitation assays for interaction between SrfB and SrfA. (**D**) Co-immunoprecipitation assays for interaction between SrfC and SrfB.

Firstly, the lysate of GB05/srfABC after being induced with L-arabinose was separated by native-PAGE and analyzed by western blotting analysis to investigate whether three components could form a heteromeric complex. All three antibodies directed against SrfA, SrfB, and SrfC reacted with a major complex (Figure 7A), suggesting that SrfA, SrfB, and SrfC could form a heteromeric complex in vivo. Moreover, anti-SrfB antibody also reacted with another complex with higher molecular weight, probably because SrfB could form a homomeric complex.

Subsequently, we performed co-immunoprecipitation experiments to investigate the interaction between each component. The immunoblots showed that in the presence of anti-SrfC antibody, SrfA protein could be co-immunoprecipitated with SrfC protein (Figure 7B). Furthermore, anti-SrfB antibody was able to bring down SrfA protein (Figure 7C), while SrfB protein could be co-immunoprecipitated with SrfC protein when anti-SrfC antibody was present (Figure 7D). The above data indicated that SrfA, SrfB, and SrfC proteins could interact with each other and form a heteromeric complex.

Despite the similarity between SrfABC toxin and other tripartite toxins in terms of interaction between components, however, it seems that SrfABC toxin employs a novel mechanism of cytotoxicity. Each component of SrfABC toxin could enter HeLa cells independently and induce cytotoxicity and apoptosis on HeLa cells with varying extents. However, in the case of CDT toxin, only COS-1 cells transfected with a plasmid expressing CdtB exhibited striking alterations in morphology, while cells expressing CdtA or CdtC displayed an apparently normal morphology and showed no signs of intoxication [15]. A combination of CdtB and either CdtC or CdtA did not result in toxicity for treated cells. CdtB alone is unable to reach the intracellular compartment to exert its toxic effect. In the case of Tc toxin, the TcA component is thought to be involved in binding and translocation of the biologically active TcC protein with the cytosol of target cells [20]. Based on the novel mechanism of cytotoxicity together with amino acid sequences and locus organization, SrfABC toxin cannot be classified in any known family of cytotoxins, which suggests that SrfABC toxin belongs to a new family of tripartite cytotoxins.

## 3. Materials and Methods

### 3.1. Bacterial Strains, Plasmids, and Cell Lines

*X. stockiae* HN_xs01 strain was grown in Luria-Bertani (LB) medium at 30 °C, and *E. coli* strains were grown in LB medium at 37 °C. *E. coli* GB05 was used as the host for DNA cloning, and BL21 was used as the host for SrfA, SrfB, and SrfC protein expression. Plasmid pSC101-BAD-gbaA, which harbors the arabinose-inducible promoter pBAD [23], was used for the *srfABC* operon expression in *E. coli* bacterial cytoplasm. Plasmid pGEX-4T-2 (GE Healthcare, Piscataway, NJ, USA) was used for individual expression of GST-tagged SrfA, SrfB, and SrfC proteins in BL21 [9]. Plasmid pET28a (Novagen, Madison, WI, USA) was used for individual expression of His-tagged SrfA, SrfB, and SrfC proteins in BL21 (DE3). Eukaryotic expression vector pcDNA5/FRT/TO/FLAG (Invitrogen, Carlsbad, CA, USA) was used for transient expression of SrfA, SrfB, and SrfC proteins in HeLa cells. All mammalian cancer cell lines, including human hepatoma cell line Hep-3B, human cervical carcinoma cell line HeLa, murine melanoma cell line B16, and murine mammary carcinoma cell line 4T-1, were cultured in RPMI 1640 medium supplemented with L-glutamine (Gibco, Paisley, UK) and 10% heat-inactivated FBS, 100 U/ mL penicillin, and 100 μg/ mL Streptomycin at 37 °C.

### 3.2. Plasmids Construction

Genomic DNA was extracted from *X. stockiae* HN_xs01 using Genomic DNA Extraction Kit (Qiagen, Courtaboeuf, France) and used as a template for PCR amplification of *srfA*, *srfB*, and *srfC* genes. PCR products of *srfA*, *srfB*, and *srfC* genes were double-digested with KpnI/XhoI and cloned into the corresponding sites of eukaryotic expression vector pcDNA5/FRT/TO/FLAG to create pFLAG–SrfA, pFLAG–SrfB, and pFLAG–SrfC, respectively. All the constructs were confirmed by DNA sequencing.

### 3.3. Sample Preparation

Overnight culture of GB05/srfABC [9] was transferred into fresh LB medium and grown to an OD_600nm_ = 0.4–0.6 at 37 °C. Then, 10 μL 10% L-arabinose was added to induce the cultures, and cells were grown at 37 °C for another 1 h. Bacterial cells were harvested and washed three times with distilled water, and then the cells were suspended in lysis buffer and lysed by sonication. Various amounts of the lysates of L-arabinose-induced GB05/srfABC were applied to mammalian cancer cell lines.

GST-tagged and His-tagged SrfA, SrfB, and SrfC proteins were purified by affinity chromatography as previously described [9]. The protein content was determined using the Bio-Rad Bradford reagent. Purified His–SrfA, His–SrfB, and His–SrfC proteins were used for antiserum production in rabbits as previously described [24].

### 3.4. Cell Viability Bioassay

The effect of different concentrations of SrfA (50–150 nM) alone, SrfB (50–150 nM) alone, and SrfC (50–150 nM) alone, and their mixture (SrfA/SrfB/SrfC = 1:1:1) on cell viability was determined for HeLa cells. Wells of a 96-well microtiter plate were loaded with 100 μL of cell suspension (2.0 × 10^5^ cells/mL) and exposed to object proteins or phosphate-buffered saline (PBS) in the control treatment. The plates were incubated for 72 h at 28 °C. Cell viability was determined by the MTT [3-(4,5-dimethylthiazol-2-yl)-2,5-diphenyl tetrazolium bromide] assay. The absorbance was read at 490 nm using an enzyme-linked immunosorbent assay plate reader. Cell morphological changes were analyzed by an inverted light microscope (Leica DMIL; Leica Microsystems S.p.A, Milan, Italy). For each concentration, three replicates were performed and each experiment was repeated twice.

### 3.5. Detection of Apoptosis

Cell apoptosis was detected with the Annexin V-FITC/PI Apoptosis Detection kit (BD Biosciences, San Diego, CA, USA) according to the manufacturer’s instructions. In brief, HeLa cells were seeded in 6-well culture plates in RPMI 1640 medium at the concentration of 2 × 10^5^ cells per well. The following day, the cells were treated with SrfA (150 nM) alone, SrfB (150 nM) alone, or SrfC (150 nM) alone, and their mixture (50 nM for each component) for a 24 h period. Cells were trypsinized and suspended in 500 μL of binding buffer containing 5 μL Annexin V-FITC and 5 μL propidium iodide (PI). After incubation in the dark for 1 h, HeLa cells were subjected to a CytoFLEX flow cytometer (Beckman Coulter), and rates of cell apoptosis were analyzed with CellQuest software. For each sample, three replicates were performed, and this experiment was repeated twice.

### 3.6. Transfection

HeLa cells were seeded in six-well plates (2 × 10^5^ cells per well), cultured overnight at 37 °C in 5% CO_2_ and grown to 60% confluence prior to transfection. Transfection with pFLAG–SrfA, pFLAG–SrfB, or pFLAG–SrfC was performed by Effectene Transfection Reagent (QIAGEN) according to the manufacturer’s instructions. HeLa cells transfected with pcDNA5/FRT/TO/FLAG served as the control.

### 3.7. Western Blot Analysis

Protein samples were separated by SDS-PAGE and then transferred onto polyvinylidene difluoride (PVDF) membranes. Afterwards, the PVDF membranes were incubated with primary rabbit antiserum (a dilution of 1:1000), and anti-rabbit secondary antibody conjugated to horse-radish peroxidase (Sigma-Aldrich Co. Ltd. Shanghai, China) was used. Binding of the secondary antibody was detected with 3, 3-diamino-benzidine tetrahydrochloride solution (DAB, Sigma).

### 3.8. Immunostaining and Confocal Microscopy

After being treated with SrfA, SrfB, or SrfC protein alone, or their mixture, HeLa cells were fixed with methanol for 10 min and permeabilized with 0.25% Triton X-100 in PBS for 10 min at room temperature. Subsequently, cells were incubated with anti-His–SrfA, anti-His–SrfB, or anti-His–SrfC antibodies (1:1000), respectively, for 2 h at room temperature and then incubated with Alexa Fluor 488-conjugated secondary antibody (Invitrogen). After being washed with PBS, cells were incubated at room temperature for 20 min in Hoechst 33342 to visualize the cell nucleus. After washing in PBS, the coverslips were mounted on glass slides with a ProLong Antifade kit (Invitrogen), and cells were examined using a Zeiss LSM 510 META confocal microscope (Germany) and processed with LSM Image Browser software.

### 3.9. Native PAGE

Native PAGE was used to analyzed the SrfABC toxin complex formed when they were simultaneously expressed in the *E. coli* bacterial cytoplasm. Lysate of L-arabinose-induced GB05/srfABC was collected, and 20 μL aliquot was analyzed by 6% native PAGE gel as previously described.

### 3.10. Co-Immunoprecipitation

To investigate reciprocal interaction between three components of SrfABC toxin, His-tagged protein (His–SrfB or His–SrfC) mixed with GST-tagged protein (GST–SrfA or GST–SrfB) was subjected to immunoprecipitation with anti-His–SrfB or anti-His–SrfC antibody. With detection of the interaction between His–SrfC and GST–SrfA, for example, both proteins were first diluted to a concentration of 1 μg/μL in IP buffer (25 mM Tris–HCl, pH 7.5, 150 mM NaCl, 0.5 % Triton X-100, 1 mM EDTA, and 1% protease inhibitor) and mixed together. Protein A/G Agarose Beads (40 μL; Abmart) were added to the mixture and incubated at 4 °C with gentle shaking for 3 h. Agarose beads were removed by centrifugation and anti-His–SrfC antibody was added to the supernatant. After overnight incubation at 4 °C, 40 μL of protein A Sepharose was added and incubated for a further 3 h at 4 °C. Beads were collected by centrifugation followed by washing four times with ice-cold IP buffer. Bound proteins were released from beads by boiling in SDS gel loading buffer for 5 min and subjected to western blotting analysis.
